# Understanding the Potential Impact of a Combination HIV Prevention Intervention in a Hyper-Endemic Community

**DOI:** 10.1371/journal.pone.0054575

**Published:** 2013-01-23

**Authors:** Ramzi A. Alsallaq, Jared M. Baeten, Connie L. Celum, James P. Hughes, Laith J. Abu-Raddad, Ruanne V. Barnabas, Timothy B. Hallett

**Affiliations:** 1 Global Health, University of Washington, Seattle, Washington, United States of America; 2 Medicine, University of Washington, Seattle, Washington, United States of America; 3 Epidemiology, University of Washington, Seattle, Washington, United States of America; 4 Biostatistics, University of Washington, Seattle, Washington, United States of America; 5 Statistical Center for HIV/AIDS Research and Prevention, Fred Hutchinson Cancer Research Center, Seattle, Washington, United States of America; 6 Vaccine and Infectious Diseases Division, Fred Hutchinson Cancer Research Center, Seattle, Washington, United States of America; 7 Infectious Disease Epidemiology Group, Weill Cornell Medical College - Qatar, Qatar Foundation - Education City, Doha, Qatar; 8 Department of Public Health, Weill Cornell Medical College, Cornell University, New York, New York, United States of America; 9 School of Public Health, Imperial College London, London, United Kingdom; Yale School of Public Health, United States of America

## Abstract

**Objectives:**

Despite demonstrating only partial efficacy in preventing new infections, available HIV prevention interventions could offer a powerful strategy when combined. In anticipation of combination HIV prevention programs and research studies we estimated the population-level impact of combining effective scalable interventions at high population coverage, determined the factors that influence this impact, and estimated the synergy between the components.

**Methods:**

We used a mathematical model to investigate the effect on HIV incidence of a combination HIV prevention intervention comprised of high coverage of HIV testing and counselling, risk reduction following HIV diagnosis, male circumcision for HIV-uninfected men, and antiretroviral therapy (ART) for HIV-infected persons. The model was calibrated to data for KwaZulu-Natal, South Africa, where adult HIV prevalence is approximately 23%.

**Results:**

Compared to current levels of HIV testing, circumcision, and ART, the combined intervention with ART initiation according to current guidelines could reduce HIV incidence by 47%, from 2.3 new infections per 100 person-years (pyar) to 1.2 per 100 pyar within 4 years and by almost 60%, to 1 per 100 pyar, after 25 years. Short-term impact is driven primarily by uptake of testing and reductions in risk behaviour following testing while long-term effects are driven by periodic HIV testing and retention in ART programs. If the combination prevention program incorporated HIV treatment upon diagnosis, incidence could be reduced by 63% after 4 years and by 76% (to about 0.5 per 100 pyar) after 15 years. The full impact of the combination interventions accrues over 10–15 years. Synergy is demonstrated between the intervention components.

**Conclusion:**

High coverage combination of evidence-based strategies could generate substantial reductions in population HIV incidence in an African generalized HIV epidemic setting. The full impact could be underestimated by the short assessment duration of typical evaluations.

## Introduction

Three decades into the fight to prevent new HIV infections, no single intervention has been found to have sufficiently high efficacy, acceptability, feasibility, and affordability to individually control the generalized HIV epidemic in sub-Saharan Africa. However, a growing number of interventions have been shown to partially reduce HIV risk and have been demonstrated to be deliverable at scale in Africa. These include reducing sexual risk behaviour by increasing condom use and decreasing partner acquisition rate, reducing the likelihood of HIV acquisition by male circumcision for HIV uninfected men [1]–[4], and reducing the infectiousness of persons with HIV by antiretroviral treatment (ART) [5], [6]. High coverage of these available, partially-effective interventions, delivered in combination, could have substantial effects on population-level HIV incidence.

HIV testing is the crucial entry point to effective HIV prevention. For infected persons, knowledge of HIV status enables referral to care and assessment for ART. Testing can also motivate male circumcision for HIV uninfected men and reductions in risk behaviour, particularly among newly diagnosed persons with HIV [7]–[10]. However, a large proportion of adults in sub-Saharan Africa do not know their HIV serostatus [11]; which impedes the delivery and uptake of combination HIV prevention intervention. Among successful strategies that have been used to greatly increase HIV testing in a community [12], [13] is door-to-door household-based testing and counselling (HBCT). HBCT programs in Uganda and South Africa have demonstrated high testing coverage, with 60–80% of adults learning their HIV serostatus [14]–[17]. In one example, community HBCT was followed by large increases in the frequency of condom use amongst HIV-infected men [8], suggesting that wide-scale testing may also shift behavioural norms.

Implementation and field evaluation of wide-scale HIV testing coupled with combination HIV prevention interventions are urgently needed. Program evaluations and clinical trials are underway [18]. In advance of results from those studies, epidemiological and theoretical analyses are required to understand how different components of the HIV combination interventions might operate at a population level and how combination interventions should be designed.

Some previous model projections have suggested that ART alone could achieve high impact on HIV incidence [19], [20], but have required extremely demanding assumptions such as universal testing every year and perfect adherence on ART that may not be attainable [21]–[27] and could be costly [28]. Also, it is unlikely that strategies based on ART alone would be enough due to the difficulty of targeting people with primary HIV infection and suboptimal adherence and retention in care [29].

In this article, we use a mathematical model of the HIV epidemic in South Africa to study HIV incidence under rapid and high testing coverage that is repeated every four years and the following interventions: risk reduction following HIV diagnosis (the first HIV-positive test), medical male circumcision, and ART initiation following current WHO guidelines (CD4 count ≤350 cells/ml). First, the interventions are considered individually to disentangle and characterize their impacts. Secondly, we estimate the population-level impact of a feasible implementation of a combination intervention and examine how trials to measure these effects might be designed. Thirdly, we studied the implications of expanding ART initiation to be upon HIV diagnosis. Finally, we determine the factors that influence this impact and quantify the interaction between the components in the combined intervention at the short and the long terms. The HIV settings of South Africa are ideal for our study because of very high HIV prevalence (17% among 15–49 year-old men and women) and because trials of a combination of deliverable prevention interventions are being planned in similar settings.

## Methods

### The Mathematical Model

**Figure 1:**
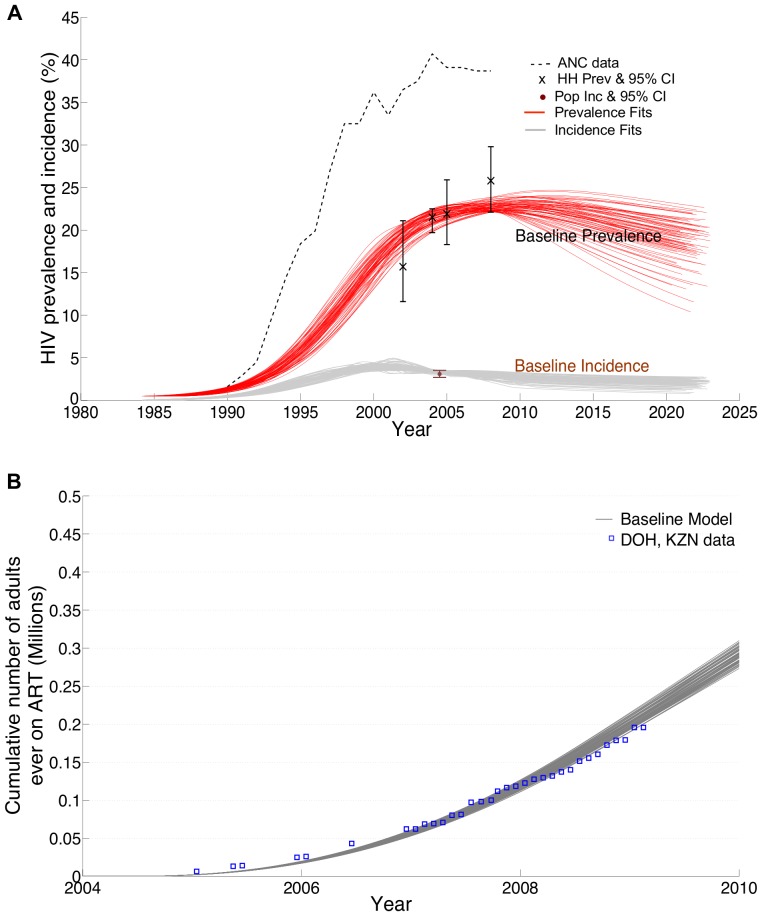
Calibration of model baseline projections by data from Kwazulu-Natal, South Africa. Sources of data used to calibrate the baseline model were in A) HIV prevalence among women attending prenatal clinics [46] (dashed lines); HIV prevalence measurement in household-based surveys (crosses) [36], [47]; estimates of HIV incidence in household-based surveys [48] (dot). In B) district-wide ART coverage statistics [49] were used to calibrate baseline trends of ART recruitment. Multiple sets of parameters that were consistent with these data were identified [72] and generated model trajectories of baseline prevalence (red lines) and baseline incidence (grey lines) a sample of which is shown here (Supplementary Material S1).

We constructed a compartmental mathematical model to represent transmission of HIV in the heterosexual adult population in KwaZulu-Natal (KZN), South Africa. A full technical specification of the model and its parameterization is given in Supplementary Material S1 and Table 1 (or Table 2 in Supplementary Material S1) lists the main model inputs. Briefly, the average sexual life span without HIV infection is 35 years while the mean duration of the untreated HIV infection is 11 years and is characterised by stages that vary by CD4 counts and infectiousness (Figure 1 in Supplementary Material S1). Infectiousness varies over the course of infection in the manner estimated by Hollingsworth *et al*. based on analyses of cohort data from rural Uganda [30], [31] (Table 1). Following others [32]–[34], the modelled population is stratified according to sex, circumcision status of men, and risk group, with the different risk groups forming new sexual partnerships at different rates. The model is fitted to HIV prevalence and incidence data from KZN, generating a prediction for the time-course of the baseline HIV incidence in KZN (Figure 1). The potential influence of the type of model structure was assessed through comparison with published model results that used different methods and predict the potential influence of expanding treatment or circumcision [19], [20], [28], [34], [35]. We did not fit the model to sex specific HIV incidence data.

**Table 1:** Main model assumptions.

Assumption	Parameter value	Sources
Demography and sexual behaviour		
**Average sexual life span**	**35 years**	**Representative of adult population of age (15–49)**
**Number of unprotected sexual acts per partnership in a partnership with low sexual activity individual**	**100**	**Based on estimated frequency of sex in marital relationships in South Africa ** **[73]**
**Otherwise**	**2**	
HIV pathogenesis		
**Mean duration of infection (untreated HIV)**	**11.0 years**	**[30]**, **[74]**–**[76]**
**Excessive mortality (when initiating ART at CD4>200) compared to non-infected persons**	**5.0 per 100 person-years**	**[77]**
**Excessive mortality (when initiating ART at CD4≤200) compared to non-infected persons**	**8.0 per 100 person-years**	**Representative for heightened morbidity in patients receiving ART at CD4<200 compared to patients starting ART at CD4>200 ** **[78]**, **[79]**
Multiplicative factor change in baseline HIV transmission probability		
**From population with acute infection**	**27**	[**75**]
**From females relative to from males**	**0.5**	**[80]**, **[81]**
**From HIV infected individuals at risk of opportunistic infections and heightened viremia in late symptomatic infection stages**	**7**	[**75**]
**From individuals with AIDS**	**0**	**Estimated**
**From circumcised men**	**1**	**No effect**
**From individuals on ART**	**0.08**	**ART efficacy of reducing onward transmission for persons on ART versus persons not on ART ** **[5]**, **[6]**
**To circumcised men**	**0.4**	**[1]**–**[4]**
**Condoms both ways**	**0.1**	**[82]**

**Table 2:** Model assumptions of efficacy and uptake for HIV testing, risk behaviour reduction, circumcision and ART in the current standard and the combination interventions.

Intervention Scenario:	*Current standard*	*“Combination I”*	*“Combination II”*
**HIV Testing**			
Nature of testing	Continuously available via VCT	Community testing	Community testing
Coverage of HV testing (% of eligible adults)	20 [36]	90	90
Interval before reaching the above coverage level (years)	none	1	1
Interval between rounds of testing (years)	no rounds	4	4
**Risk Behaviour reductions following HIV diagnosis (Relative to being unaware of status)**			
Increase in condom use (men/women)	12.5%/6.25%§	25%/12.5%	25%/12.5%
Decrease in partner acquisition rate (men/women)	12.5%/12.5%§	25%/25%	25%/25%
Mean duration of keeping the above behaviour changes	1 year§	3 years	3 years
**Circumcision Intervention**			
Circumcision effect reducing men’s susceptibility to HIVper sex act	60% [1]–[3]	60% [1]–[3]	60% [1]–[3]
Proportion of uncircumcised men that are newly circumcised (final levels are continued in the future)	10% over 4 years	70% over 2 years	70% over 2 years
**ART initiation**			
ART efficacy of reducing infectiousness	92% [5]	92% [5]	92% [5]
CD4 count threshold at which tested individuals start ART	200^*^	350**	any CD4 count**
Coverage	52% [43]	100%	100%
Drop out rate (per year) from ART initiated at any CD4 count	14.5% [44], [84]	14.5% [44], [84]	14.5% [44], [84]

§ Representative values [83], Baseline values of condom use are listed in Table S3 in Supplementary Material.

** Treatment initiation at HIV stages with CD4>100 cells/mm^3^ is immediate when the individual is aware of status and meeting the guideline and is delayed when unaware of status by an average duration that is calibrated to reflect current treatment programs [37], [38].

The intervention scenarios investigated focused on three partially-effective HIV interventions and were based on achieving 90% HIV testing of adults over one year (for example through community home based counselling and testing (HBCT)) and repeated every four years (in the text below we refer to this as ‘community testing’). The three interventions are –1) Risk behaviour reduction for individuals newly diagnosed with HIV, 2) male circumcision for HIV uninfected men, and 3) ART initiation for HIV infected persons. A combination prevention intervention consists of the effects of these three interventions acting together in KZN population and we studied two possible combination interventions (Table 2). Estimated HIV incidence values under individual and combined interventions are compared against the HIV incidence under a scenario representing the current standard of care (Table 2). The *current standard scenario* in Table 2 reflects the status quo in South Africa where 20% of the population is tested for HIV annually [36], 10% of men undergo medical circumcision in 4 years, and ART threshold of initiation is according to former South Africa guideline at CD4 count ≤200 cells/mm^3^ but most treatment initiation occurs at CD4 count <100 cells/mm^3^ due to the low rate of testing [37], [38]. Also in this scenario men and women newly diagnosed with HIV increase condom use by 12.5% and 6.25% and decrease partner acquisition rate by 12.5% and 12.5%, respectively, and keep this reduction in risk behaviour for a year on average.

Assumed coverage levels of the community testing and the interventions were chosen to be optimistic but potentially achievable. Community testing increases the proportion of adults that ‘know’ their HIV status to 90%; new rounds of community testing are implemented every 4 years, allowing persons infected in the interim or not tested in previous rounds to learn their status. No testing is assumed to take place between the rounds of the four-yearly community testing and sensitivity analyses incorporated lower testing levels in each HIV testing. The model allows retesting individuals and tracks whether they have been infected since their last HIV test or remain uninfected (Figure 2 in Supplementary Material S1). HIV-infected adults who are newly diagnosed, unless otherwise noted, experience risk behaviour changes by increasing their frequency of condom use and reducing formation of casual partnerships [8], [39]. We assumed no reduction in risk behaviours for persons who test HIV negative. Newly diagnosed HIV-positive men and women increase condom use by 25% and 12.5% and a decrease in partner acquisition rate by 25% and 25% for an average duration of 3 years, respectively. A spectrum of risk behaviour reduction with variable duration was studied for sensitivity analysis.

**Figure 2:**
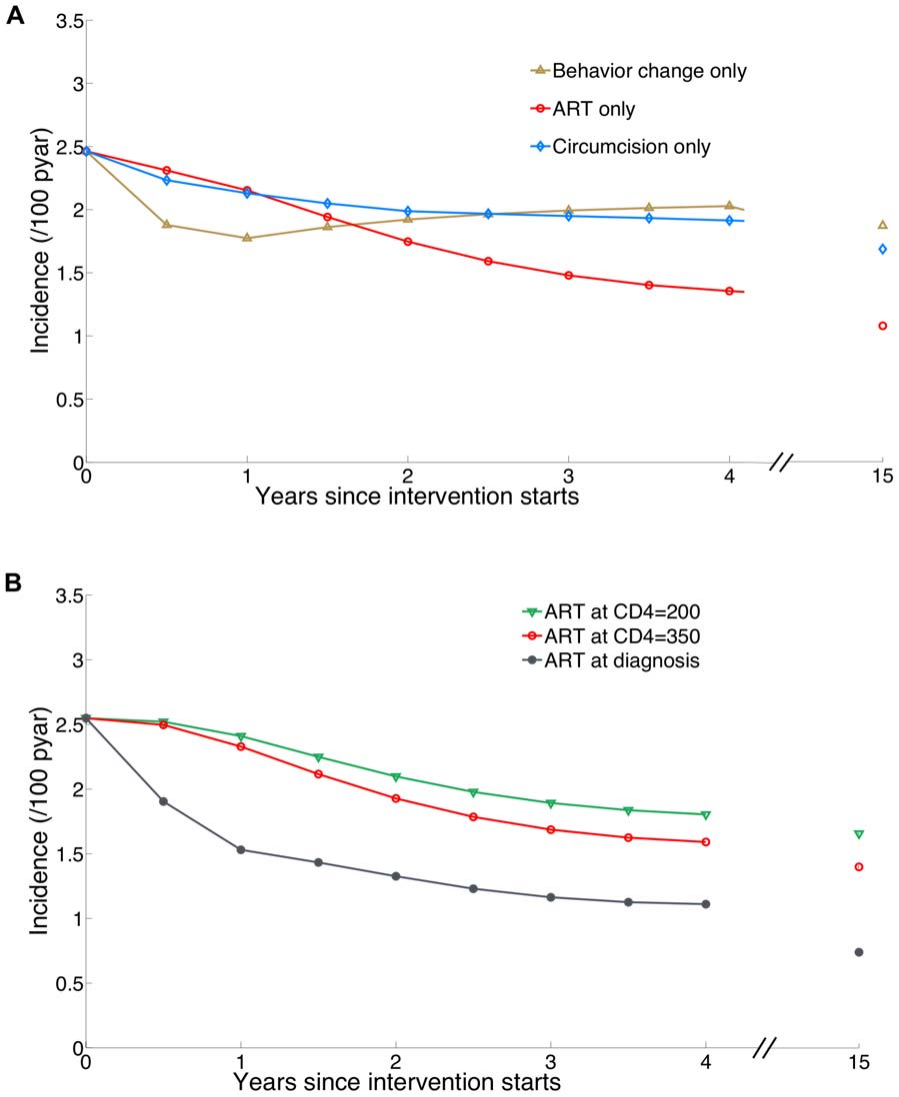
Projections of HIV incidence under implementations of single intervention components after one round of community HIV testing. (A) The individual impacts of risk reduction following HIV diagnosis (behaviour change), ART, and circumcision acting alone as single intervention components. ‘ART only’ designates initiating treatment at CD4 threshold of 350. (B) HIV incidence after implementation of ART initiation at CD4 count threshold of 200, 350, and at any CD4 count for all individuals that have been tested. All Interventions are assumed to commence in 2014. In this figure we assume counterfactually that individuals newly diagnosed with HIV do not reduce risk behaviour except in panel (A) in which the individual impact of risk reduction following HIV diagnosis is studied. Projections at year 15 are also displayed for comparison.

Current KZN circumcision prevalence (27% [40]) is assumed in all model simulations as the circumcision rate at adolescence. Consistent with South African national targets for 2016 [41], circumcision intervention is represented by a proportion (70%) of uncircumcised men getting circumcised over 2 years from the start of the intervention campaign with sufficient circumcision operations in subsequent years to sustain this level of coverage. Lower circumcision levels of coverage are considered in sensitivity analyses. Male circumcision is assumed to reduce the chance of female-to-male transmission by 60% in each sex act [1]–[3].

HIV infected individuals who are aware of their HIV status are implicitly assumed to have regular CD4 count monitoring and thus access ART promptly after their CD4 cell count falls below a chosen threshold (two thresholds are modelled: former South Africa guideline to initiate ART at CD4 ≤ 200/mm^3^ and current South Africa and WHO guidelines, to initiate ART at CD4 ≤ 350 cells/mm^3^ [42], as well as ART soon after their first positive HIV test, irrespective of CD4 count). Individuals unaware of their HIV status typically initiate ART according at CD4 count below 100 cells/mm^3^. Individuals receiving ART stop doing so (‘drop out’) at a rate of 14.5% per year [43] which corresponds to reported ART retention in South Africa [44]. HIV-infected individuals on ART are assumed to be 92% less infectious than individuals not on ART [5], [6].

Measuring the interaction between co-existing interventions: to tease out any interaction between the interventions *1,2,3, …,n* when their effects coexist in a population (that is to say they are combined) we introduce a measure of synergy through calculating extra reduction in incidence. Let the incidence under current-standard at time *t* be *I_0_(t)* and the incidence under the individual interventions *1, 2, 3, …, n* be *I_1_(t), I_2_(t), I_3_(t),…, I_n_(t),* respectively. If the incidence when the interventions are acting together is *I_c_,* then:
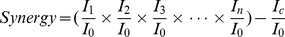
(1)


The combined interventions have interaction if *Synergy* is non-zero, have synergy if *Synergy* is positive, and otherwise they are redundant: that is one or more of the components are preventing what the rest of the components are already preventing. To be noted here is that the above synergy measure is a time varying quantity because incidence varies with time.

### Model Analysis

First, we disentangle the time variation of the impact of the three interventions mentioned above by having them acting alone in the population of KZN. Secondly, we estimate the joint impact when all of these interventions are in action (denoted as the “Combination I” intervention in Table 2) and quantify the influence of key determinants of the short-term (at 4 years) and longer-term (at 15 years) impact on population HIV incidence. Thirdly, we examine the additional impact of extending the combination intervention to include treatment upon HIV diagnosis irrespective of CD4 cell count – termed the “Combination II” intervention. Model assumptions for these interventions are summarized in Table 2. Finally, we study the interaction between the interventions in Combination I and Combination II over time.

## Results

### 1. Model Fitting

An uncertainty range for the key results is presented below between parentheses to reflect the uncertainties in calibrating model parameters to the epidemiological context of KZN. To reflect this uncertainty, model parameters that are difficult to estimate reliably are randomly selected, using prior distributions, from within credible limits based on local demographic data, reported indicators of sexual behaviour (Table 1 in Supplementary Material S1). This translates the uncertainty in the model parameters to multiple model projections. Following a rejection-sampling procedure [45] (Supplementary Material S1), we kept only model projections consistent with: measurements of HIV prevalence among women attending prenatal care [46] and in household surveys [36], [47], HIV incidence point estimate using longitudinal HIV surveillance data from KZN [48] (Figure 1(A)), and province-wide ART coverage statistics [49] (Figure 1(B)). Thus, the uncertainty range of a scenario is obtained by running the scenario over alternative model fits (Figure 1) and evaluating the interquartile range of the results.

### 2. Individual Impact of Component Interventions

Here, we examine the impact of the individual interventions on HIV incidence at an early time point (four years after initial implementation); (Figure 2(A)). Risk behaviour reduction following HIV diagnosis would be expected to have the greatest impact in the first years following community testing, but its impact would then wane, because this reduction in risk is not sustained. The impact of circumcision grows over time, as more men become circumcised and receive direct protection from HIV infection, and as women begin to receive an indirect protection through lower HIV prevalence in their male partners. In KZN, the impact of male circumcision as a single intervention is limited (compared to other settings) given the baseline 27% prevalence of circumcision among men [40].

To disentangle the effect of ART from risk behaviour reduction among newly-identified HIV-infected adults (in Figure 2), we assume counterfactually that individuals newly diagnosed with HIV do not reduce risk behaviour. The impact of ART alone, initiated at CD4≤350 cells/mm^3^ on HIV incidence is initially small but grows quickly as a substantial proportion of HIV-diagnosed adults initiate ART and then reach later stages of infection while being less infectious than they otherwise would be [5]. Among the individual interventions based on community testing, ART as a sole intervention achieved the greatest impact; by 4 years, HIV incidence was reduced by 33% (32%–34%) (from 2.3 (2.1–2.6) per 100 person-years (pyar) under the current standard to 1.6 (1.4–1.7) per 100 pyar).

Different thresholds for ART initiation upon knowledge of status were explored further (Figure 2(B)). Compared to the current standard, in which low testing rates lead to late entry to care and treatment initiation at CD4 cell counts mostly below 200 cells/mm^3^ (Table 2), the high community testing rates and ART initiation would generate progressively greater benefits if started at higher CD4 counts, particularly if ART is initiated upon HIV diagnosis. ART initiation at CD4 counts ≤350 cells/mm^3^ slightly improves the impact compared to ART at ≤200 cells/mm^3^ (33% (32%–34%) versus 24% (23%–25%) reduction in HIV incidence at 4 years). In contrast, with immediate ART initiation upon diagnosis the impact on HIV incidence would be more substantial; our model predicts a 53% reduction in incidence at 4 years compared to the current standard; growing to 66% at 10 years and 67% at 15 years (Figure 2(B)). The greater initial and long-term impact of ART initiation upon diagnosis is due to substantially more individuals readily starting ART, including some in the highly infectious early phases of infection, with their infectiousness immediately reduced. For all ART initiation strategies, the full prevention benefits would not be achieved until 10–15 years after the start of intervention, when averted infections have terminated chains of further transmissions. However, for this ‘ART-only’ projection the model does not project HIV incidence to ever fall below 0.5/100 pyar. This estimate is higher than other model estimates for ‘Universal test and treat’ intervention [19], [28], because of the partial reduction in HIV infectivity assumed for ART users [5], [50] and due to assumptions about the drop-out from ART programs and the suboptimal testing coverage of the four-yearly community testing leading to delays in initiating ART. The projections of incidence under individual interventions illustrate the levels of coverage required for large impact to be attained.

### 3. The Impact of a Combination Intervention

**Figure 3:**
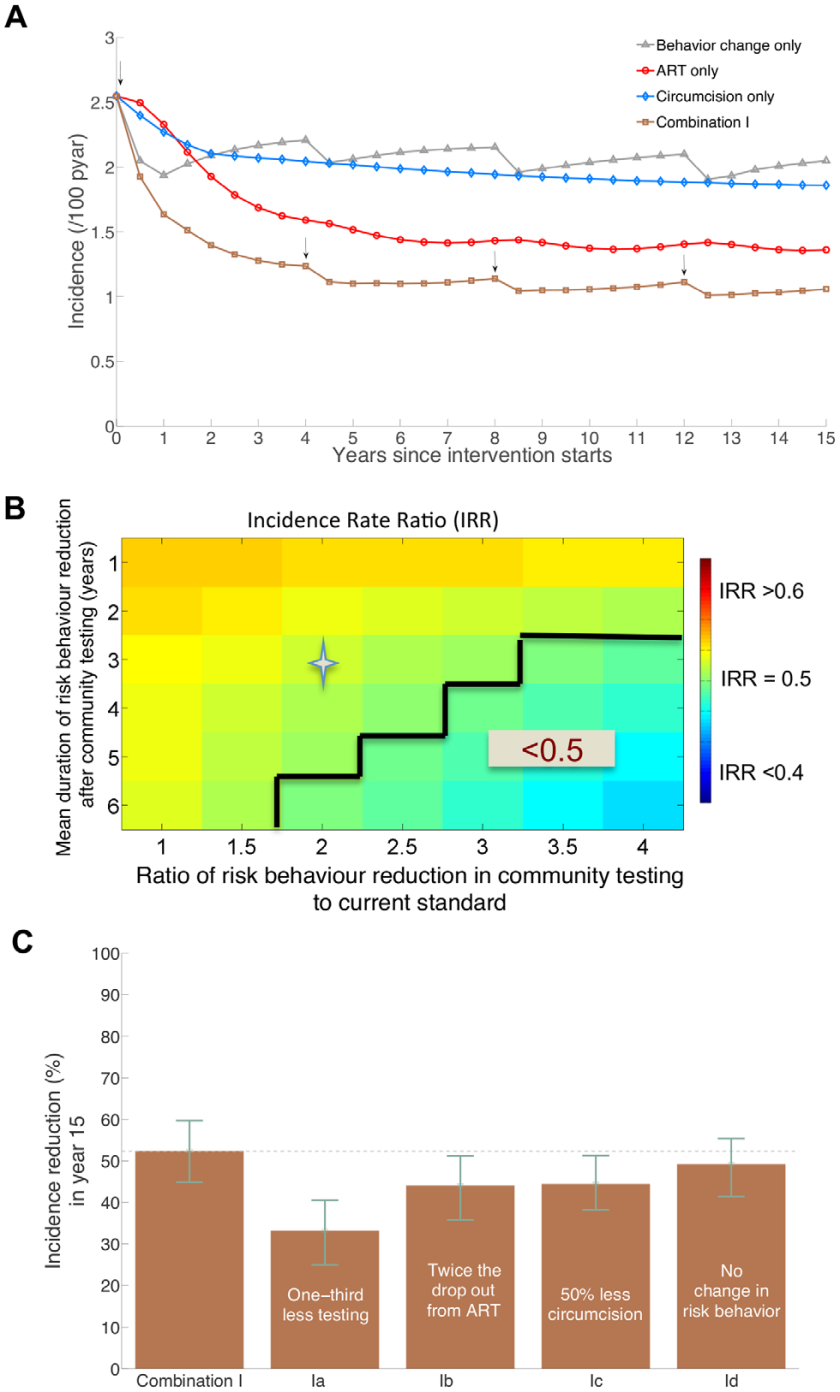
Projections of the impact of Combination I intervention on HIV incidence. (**A**) A 15-year incidence projections with the continuation of the intervention (arrows indicate the rounds of community HIV testing which are every four years) compared to incidence projections under individual intervention components (**B**) Projected incidence rate ratio (IRR) (colour coded) in year 4 with respect to current practice incidence at different assumptions of risk behaviour reduction following HIV diagnosis in the community testing relative to the risk behaviour reduction in the current standard and average duration of sustainability of this behaviour change. A ratio of risk behaviour reduction of 1 indicates equal behaviour change among newly HIV-diagnosed adults in community and in venue (current standard) testing. Incidence reductions of more than 50% (IRR<0.5) are delineated and the star marks IRR value with risk behaviour change assumptions as in (A) and Table 2, (**C**) Projections of percentage reduction in incidence of the Combination I intervention with respect to current standard incidence projection of 2.1 (1.9–2.3) per 100 pyar at year 15 with various assumptions of failure to achieve the uptake levels of the interventions in Table 2: (Ia) decreasing testing uptake to 60% in community testing rounds, (Ib) doubling the drop out rate on treatment to 28% per year, (Ic) halving the uptake of circumcision to 35% of uncircumcised men, and (Id) assuming no risk behaviour change following HIV diagnosis generated by testing.

Next, we examine the impact of implementing all three components (reducing risk behaviour, male circumcision, and initiating ART at CD4 count of 350) in a combination intervention that is based on community testing (‘Combination I’ in Table 2). The model indicates that the combination intervention could reduce incidence, more than any individual intervention component simulated in Figure 2(A). At 4 years from the start of the combination prevention intervention incidence is reduced by 47% (43%–50%) to 1.2 (1.1–1.3) new infections per 100 pyar (Figure 3(A)). With the periodic rounds of community testing coupled with the interventions, the impact is maintained and strengthened; otherwise the proportion that know they are infected (and thus able to be on ART and/or maintain reduced risk behaviours) declines, as additional people become infected. If the community testing and the levels of the interventions are continued, the HIV incidence rate is expected to eventually reach 0.96 per 100 pyar at 25 years, representing a reduction in incidence of 59% compared to the incidence rate when the intervention starts.

Figure 3(B) indicates the sensitivity of the short-term impact on HIV incidence to the assumptions about the magnitude and duration of risk reduction by individuals newly diagnosed with HIV. Achieving a reduction in incidence of more than 50% in 4 years requires a 40% increase in condom use and a 40% decrease in the rate of forming new partnerships sustainable for 4 years on average, this represents more than three times the current levels and would imply significant change in community norms. If risk reduction upon HIV diagnosis amounted to a tripling in the frequency of condom use and in the rate of formation of casual partnership and lasted for six years on average, then the impact of the entire combination package would be to reduce HIV incidence by 53%. Without those risk reductions, the same package would reduce incidence by only 43%. Thus, the short-term impact of the combination intervention is most sensitive to the assumptions about the risk behaviours following the community level HIV testing campaign; shortly after the start of the intervention, the full effects of the circumcision and ART components have not accrued.

Figure 3(C) shows how the long-term impact of Combination I is affected by the failure to fully achieve the assumed coverage and efficacy levels for its components (Table 2). The long-term impact of Combination I at 15 years after the intervention starts is 52% (45%–60%) reduction in incidence (from 2.1 (1.9–2.3) to 1.0 (0.9–1.1) new infections per 100 pyar). With one-third less HIV testing in each round of community testing, reducing the awareness of status among the HIV infected individuals, the impact of the combination intervention is substantially reduced from 52% (45%–60%) to (33% (25%–41%); largely because ART initiation would be decreased. By doubling drop-out from ART, the impact of the combination intervention would also be reduced, to 44% (36%–51%). Similarly a 50% reduction in the number of men being circumcised would reduce the impact of Combination I on HIV incidence to 44% (38%–51%). The long-term impact of the combination intervention would drop by 12 percentage points (from 52% to 40%) if 10% of men compared to 70% were circumcised in 2 years (Figure 3 in Supplementary Material S1). In contrast, the long-term impact of the intervention is least affected by risk reduction following new HIV diagnosis; the long-term impact of the combination intervention is driven primarily by ART and circumcision components.

### 4. The Impact of Universal ART as Part of a Combination Intervention

**Figure 4:**
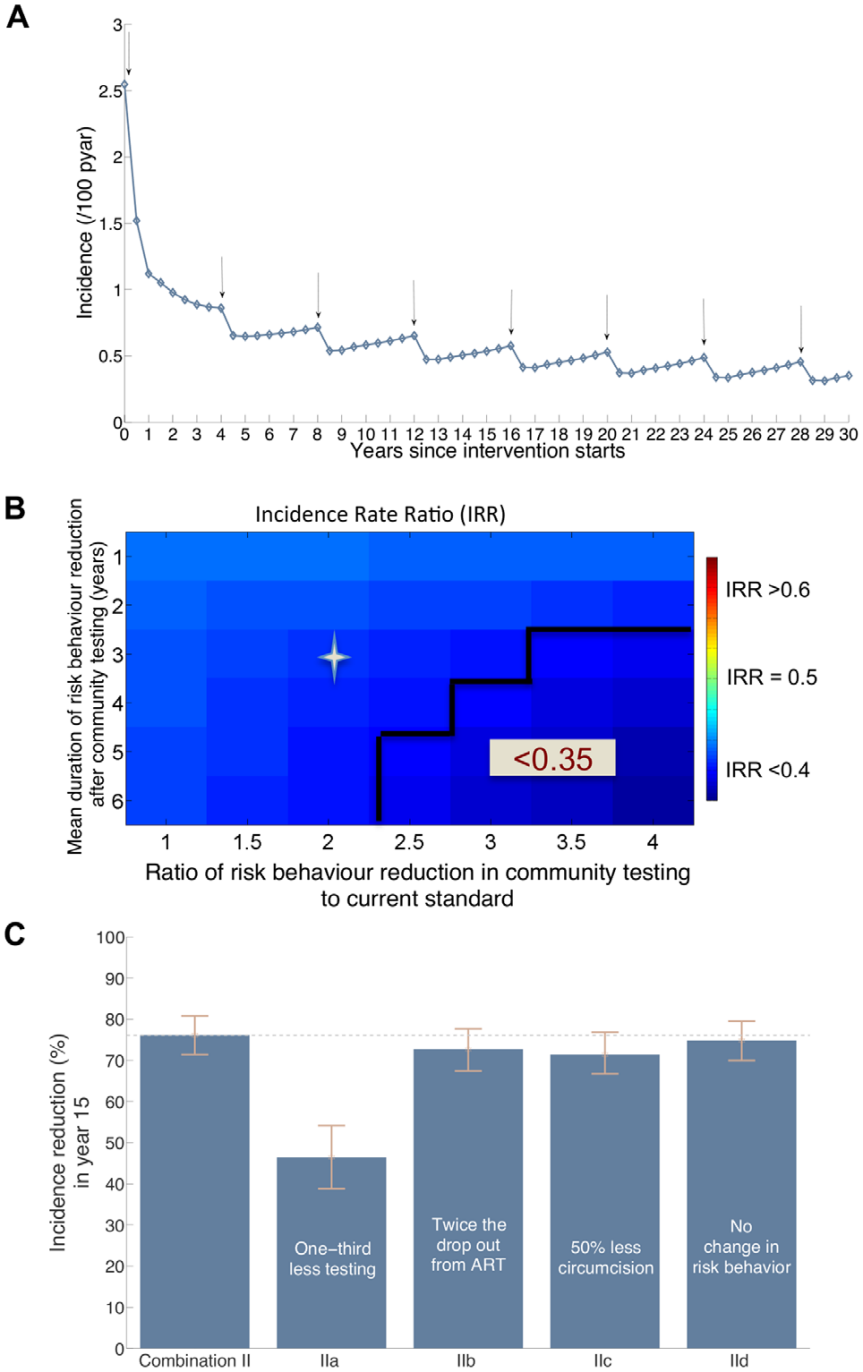
Projections of the impact of Combination II intervention on HIV incidence. (**A**) A 15-year incidence projections with the continuation of the intervention (arrows indicate the rounds of community HIV testing which are every four years). (**B**) Projected Incidence rate ratio (IRR) (colour coded) in year 4 with respect to current practice incidence at different assumptions of risk behaviour reduction following HIV diagnosis in the community testing relative to the risk behaviour reduction in the current standard and average duration of sustainability of this behaviour change. A ratio of risk behaviour reduction of 1 indicates similar extent of behaviour change among tested HIV-infected adults in community and in venue (current standard) testing. Incidence reductions of more than 65% are delineated and the star marks IRR value with behaviour change assumptions as in (A) and Table 2 (**C**) Projections of percentage reduction in incidence of the Combination II intervention with respect to current standard incidence projection of 2.1 (1.9–2.3) per 100 pyar at year 15 with various assumptions of failure to achieve the uptake levels of the interventions in Table 2: (IIa) decreasing community testing uptake to 60% in community testing rounds, (IIb) doubling the drop out rate on treatment to 28% per year, (IIc) halving the uptake of circumcision to 35% of uncircumcised men, and (IId) assuming no risk behaviour change following HIV diagnosis generated by the testing.

Finally, we investigate the impact of a combination intervention that includes expanded access to ART by initiating ART upon diagnosis (the ‘Combination II’ scenario). Under this scenario, the reduction in incidence would be greater than under Combination I at the short and long terms. At 4 years the reduction in incidence is 63% (60%–65%) versus 47% (43%–50%) for Combination I, and at 15 years the reduction in incidence is 76% (71%–81%) versus 52% (45%–60%) for Combination I (Figure 4(A)). A comparison between Figure 4(B) and Figure 3(B) shows that Combination II impact immediately after the first round of testing is less sensitive to risk behaviour reduction than the impact of Combination I. Even with risk behaviour reduction as in the current standard, Combination II is projected to achieve a 61% reduction in incidence at 4 years. Similar to Combination I, the long-term (predicted at year 15) impact of Combination II is highly sensitive to reduced uptake of HIV testing: with one third less HIV testing in each round of community testing, incidence would be reduced by 46% (39%–54%) instead of 76%. The long-term impact of Combination II is less sensitive to other assumptions about uptake of circumcision and degree of risk behaviour reduction (Figure 4(C)). The long-term impact of the combination intervention would drop by 8 percentage points (from 76% to 68%) if 10% of men compared to 70% were circumcised in 2 years (Figure 3 in Supplementary Material S1). The influence of drop-out rate from ART is small because the model allows for individuals that cease treatment after initiating at a CD4 cell count >350 cells/mm^3^ to re-initiate treatment when their CD4 cell count declines below 350 cells/mm^3^.

The continuation of HIV community testing, immediate initiation of ART, and circumcision in Combination II intervention would achieve two major goals of HIV prevention interventions. First, the rate of new HIV infections would eventually be reduced to a low level: 0.3 new infections per 100 pyar after 25 years. Second, it would reduce the rate of HIV deaths and simultaneously bring the rate of new HIV infections below the rate of AIDS death - a point defined as the ‘AIDS Transition’ [51] within 6–8 years. The trajectory of the HIV epidemic would continue to decline, potentially enabling the scale of intervention effort to eventually be reduced.

### 5. The Interaction between the Intervention Components of the Combination Interventions

**Figure 5:**
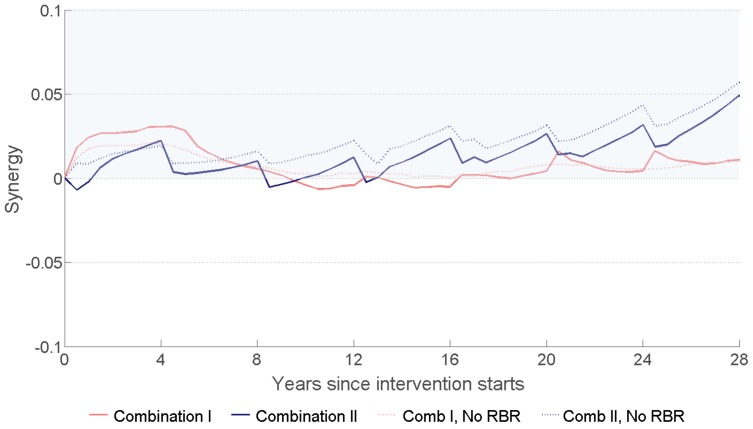
Calculated values of the synergy measure (‘*Synergy’,* ***Equation 1*** *)* indicating the interaction between circumcision, ART, and risk behaviour reduction following HIV diagnosis in Combination I and Combination II. To show the synergy between ART and circumcision, the effects of risk behaviour reduction following HIV diagnosis (RBR) are ignored in calculating the dotted lines. *Synergy* values that are larger than zero (shaded region) indicate positive interaction between the interventions and that they are complementing each other in preventing HIV transmission, while less than zero values indicate that the interventions have redundancy in prevention.

At high levels of coverage for the interventions, the interaction among them could lead to unneeded excessive protection; this is the case for example when large numbers of individuals are exposed to two interventions that reduce the same risk. Newly diagnosed individuals with HIV can be exposed to ART and risk reduction. Figure 5 shows that without risk behaviour reduction after HIV diagnosis, circumcision and ART in Combination I and II have synergy at all times. With a reduction in the risk behaviour after HIV diagnosis, the combination interventions have short periods of marginal redundancy before year 20 due to the accumulation of large numbers of individuals who have reduced risk behaviour and initiated ART. Over time, ART drop out and the waning of behavioural risk reduction eliminate such redundancy and bring back synergy among the intervention components. At the longer term, and by continuing the interventions, the substantial reduction in the rate of new infections reduces the likelihood of such redundancy.

## Discussion

We mathematically modelled a combination prevention package based on the platform of testing 90% of adults in KwaZulu-Natal, South Africa through community HIV testing campaigns every four years coupled with risk behaviour reduction following HIV diagnosis, ART initiation for HIV-infected adults according to current WHO and South African guidelines at CD4 ≤ 350 cells/mm^3^, and male circumcision for HIV-uninfected men. The model is parameterized to the epidemiological context of KZN and includes important pragmatic assumptions about initiating treatment based on knowledge of HIV serostatus, repeating community testing every four years to identify newly-infected adults and those not previously tested, and assuming realistic drop-out rates from ART, suboptimal viral suppression due to imperfect adherence on ART, incomplete uptake of circumcision with coverage consistent with targets in the South Africa’s national plan, and modest and temporary changes in sexual risk behaviours following HIV testing [5], [50]. With high levels of coverage, our analyses suggest that population HIV incidence could be reduced by almost 50% within 4 years.

The combination of intervention components that act over different time scales with different impact on HIV incidence. The combined effects of these interventions reduce both the risk of acquisition and transmission of HIV, and could generate rapid, sustained, and substantial reductions in HIV incidence. Further, our results suggest that high coverage HIV testing every four years coupled with high uptake of circumcision and ART initiation upon HIV diagnosis could reduce HIV incidence and death rates to as low as 0.3% per year, significantly reducing the scale of the epidemic. By utilizing a measure for synergy we showed that although the effects of circumcision, ART, and risk behaviour reduction coexist in the population as a result of the combination intervention, the effects substantially strengthen each other particularly if the assumed levels of coverage for these interventions are maintained.

These findings have important implications for HIV prevention prioritization and program planning. Large reductions in HIV incidence in high prevalence settings in sub-Saharan Africa can be attained with ambitious, but feasible, assumptions about scale-up coverage and impact of HIV testing, male circumcision, and ART initiation and retention. Our assumed ambitious levels of coverage are premised on high coverage in pilot work and preliminary studies; HIV testing rates in large scale HBCT programs in Uganda and South Africa have exceeded 80% [14], [17], [52]. Although in HBCT and couples counselling interventions, substantial changes in risk behaviour are observed [7], [8], [39], the projections of long-term impact of combination interventions in our model do not rely heavily on risk-behaviour change assumptions. A challenging key assumption of our model is high linkage rates of newly identified HIV-infected persons to HIV care and ART initiation, and subsequent high levels of retention in care and adherence to treatment which have been shown, consistent with our findings, to be critical for a long-lasting substantial impact of treatment at the population level [53]. A pilot of HBCT with linkages to ART in Uganda has demonstrated that active follow-up of HIV-infected persons after HBCT can achieve high linkage with 89% of HIV-infected individuals successfully referred to HIV clinics [54]).

We showed that maintaining high coverage levels of testing and risk reduction, circumcision, and HIV treatment would not compromise the synergy between these interventions. Optimizing the prevention benefits of each and every intervention of these to combat HIV is important due to the imperfections in the current HIV population-level intervention programs such as the suboptimal coverage and adherence levels, the drop out from ART, and the temporary nature of the reduction in sexual risk behaviour after HIV diagnoses.

We have not incorporated the impact of circumcision provision to HIV-infected men, because there is little evidence of direct effect of circumcision on HIV transmission to women [55], [56]. We have not included risk compensation associated with ART initiation; because several studies of sexual activity of individuals initiating ART suggest that such risk compensation is limited [57]–[59].

Although there have been other model projections suggesting single HIV interventions could achieve similar levels of impact, these have required more demanding assumptions such as universal testing every year and perfect adherence on ART that may not be consistently attainable [21]–[27]. It is unlikely that strategies based on ART alone would be enough due to the difficulty of targeting people with primary HIV infection which some models suggest account for a substantial minority of new HIV transmissions [60], suboptimal adherence to ART and follow up care [29], [61], [62].

The rapid increase in the numbers of individuals in need of HIV care and circumcision services, in Combination I and II, demands parallel increases in the capacity and the accessibility of HIV care and circumcision clinics. A parallel increase in active follow up would also be required to achieve ART timely initiation, high circumcision uptake, and the sustainability of reduced risk behaviours. The waiting period between the four-yearly testing waves might facilitate task shifting to active follow up of persons newly diagnosed with HIV. We have not investigated the financial obligations and implications of any of our scenarios. Recent studies have illustrated that among non-traditional counselling and testing methods, door-to-door HIV counselling and testing in Ugandan population has the lowest cost [16] and other studies have illustrated the cost-effectiveness of starting ART at the new WHO guideline [63]. We have shown that the intervention components in Combination I and Combination II have positive and increasing synergy at the long-term and produce extra reduction in incidence compared to when they are acting alone. Other models have also shown that multiple interventions acting together could drive greater reduction in HIV incidence than could be feasible with single interventions [64]–[66].

Our findings have significant implications for studies that aim to measure the impact of combination HIV prevention. First, the full impact of combination prevention interventions will be achieved over 10–15 years. Because most forms of impact evaluations of combination HIV prevention interventions, including community-randomized trials and programmatic assessments, are anticipated to examine impact over a much shorter period (e.g. 2–3 years), those studies could underestimate the full benefit of combination packages. Second, our model found that a determinant of short-term impact of a combination package is risk behaviour reduction following HIV diagnosis as a product of community testing. If the degree of risk reduction is less than we assumed, the ability to measure the impact on HIV incidence in a short-term evaluation is reduced. The time to see different effect magnitudes for individual components is an important consideration in the duration and interpretation of community-randomized trials of combination prevention, and argues for longer periods of observation, and monitoring intermediate biologic markers such as community viral load and key process outcomes such as number of men circumcised. The growing difference between the impacts of Combination I and Combination II over time indicates the importance of longer periods in comparing the effectiveness of different combination prevention packages. Lastly, the small relative differences in the impact of single interventions at the short-term (Figure 2(A)), could make step-wedge designs untenable for impact evaluation of separate interventions or joint effects of combined interventions (in community randomised control trials, cRCT). To successfully evaluate these effects, it would be essential to consider long intervals between the phasing of intervention components, very large numbers of communities, and large sample sizes.

Although the efficacy of topical or oral pre-exposure prophylaxis (PrEP) in reducing HIV risk has been demonstrated [67], [68], we have not included PrEP in our analysis pending further research illustrating its deliverability in sub-Saharan African settings. If PrEP is included it could strengthen the impact of a combination intervention even further.

The goal of this model was to evaluate the population-level impact on HIV incidence if high coverage levels of available strategies are achieved in KZN, South Africa. The numerical estimates presented are specific to KZN, and the impact of interventions in different contexts might vary. In settings where a lower proportion of men are already circumcised, the impact of the combination intervention could be greater and more sensitive to the uptake of circumcision. We have incorporated the uncertainty in identifying the epidemiological context of KZN using a rejection-sampling approach. Prioritizing interventions for specific age, gender, or demographic group was not the focus of this modelling exercise. Therefore, the model is not age-structured and has no details about how sexual partnerships are formed between members of different demographic groups [69]–[71]. The model does not include explicit representations for overlapping sexual partnerships. It does not account for the spread of HIV-resistant strains, or for potential changes in behaviour in the wider population following reductions in incidence and AIDS deaths. The effects of these factors can be addressed by further modelling as data become available on uptake, behaviour change, viral suppression, resistance, and clinical outcomes of earlier ART initiation.

In summary, combination HIV prevention represents the best hope for a significant impact in reducing population HIV incidence in hyperendemic countries. These theoretical model results indicate that achieving high coverage of evidence-based combination HIV prevention interventions will have a greater population impact than any of the individual interventions, and could lead to large reductions in population HIV incidence in four years with increased impact thereafter. It is now time to implement this combination prevention approach in a high prevalence sub-Saharan setting, with a rigorous evaluation conducted with sufficient coverage levels and duration in order to assess the impact on population HIV incidence.

## Supporting Information

Supplementary Material S1 (DOC)
