# Supplementary material for: Understanding the Potential Impact of a Combination HIV Prevention Intervention in a Hyper-Endemic Community
Source: PLoS One. 2013 Jan 23;8(1):e54575. doi: 10.1371/journal.pone.0054575 (PMC3553021; doi:10.1371/journal.pone.0054575)
Supplement: Supplementary Material S1 — (DOC) [file pone.0054575.s001.doc]

**Supplementary Material**

# Understanding The Potential Impact of a Combination HIV Prevention Intervention in a Hyper-Endemic Community

*Ramzi A Alsallaq^1^, Jared M Baeten^1,2,3^, Connie L Celum^1,2,3^, James P Hughes^4^, Laith J Abu-Raddad^5,7,8^, Ruanne V Barnabas^1,2,6^ & Timothy B Hallett^9^*

*^1^Global Health, ^2^Medicine, ^3^Epidemiology, and ^4^Biostatistics, University of Washington, Seattle, United States of America; ^5^Statistical Center for HIV/AIDS Research & Prevention and ^6^Vaccine and Infectious Diseases Division, Fred Hutchinson Cancer Research Center, Washington, Seattle, United States of America; ^7^Infectious Disease Epidemiology Group, Weill Cornell Medical College - Qatar, Qatar Foundation - Education City, Doha, Qatar; ^8^Department of Public Health, Weill Cornell Medical College, Cornell University, New York, New York, United States of America; ^9^School of Public Health, Imperial College London, London, United Kingdom.*

**Overview**

The mathematical model is designed to represent heterosexual HIV transmission at the *population level* in a mature generalised HIV epidemic, such as the one in Kwazulu-Natal province in South Africa, and the impact of interventions including ART, testing, change in behaviour, and male circumcision, operating either in isolation or in combination. This approach affords sophisticated insights into how these interventions could affect rates and patterns of HIV transmission, and how combinations of different forms of interventions could operate together. Key model outputs are the rate (or number) of new HIV infections across the population over time, and projections can be generated over an extended period following intervention.

**Model Structure**

The model population is divided into compartments that are differentiated by infection stage, gender, sexual behaviour and exposure to different types of interventions, with events (infection, testing, death, etc) represented as movement between these compartments . The model is population-based and deterministic, meaning that individuals and partnerships are not explicitly tracked, and that the results approximately hold only for large populations.

In the model, the course of untreated HIV infection is divided into stages (Figure S1). Individuals becoming infected move from the “Susceptible” compartment to spend average durations in consecutive compartments characterised by different CD4 counts, particularly high infectiousness, or the appearance of AIDS defining symptoms. These are: acute infection (Figure S1 ,“Acute infection”), when CD4 count is above 350 cells/mm³, when CD4 count is between 350 and 200 cells/mm³, when CD4 count is below 200 cells/mm³ and the infection is asymptomatic (Figure S1 , “Late infection”), when AIDS defining symptoms start to appear (Figure S1 , “Pre-AIDS”), and full-blown AIDS. Infectiousness of infected individuals is assumed to vary over the course of infection, including a peak of infectiousness in the “Acute infection” phase and a period of heightened infectiousness in the “Pre-AIDS” phase .

Heterogeneity in sexual behaviour is incorporated into the model by subdividing the population into strata according to their average effective partnership formation rate. Those in the higher risk groups tend to form more partnerships, but each of these partnerships comprises fewer sex acts and higher condom use. The distribution between these strata and the mean partnership change rates for men and women is estimated through reported sexual behaviour and through fitting the model to HIV prevalence data and prior information through rejection sampling approach .

**Figure S1:** Flow diagram showing model representation of HIV disease progression without (inside the oval) and with antiretroviral treatment. Entry into the sexually active population is indicated by a large black arrow. Black arrows indicate progression through untreated HIV infection stages and the likelihoods of getting ART at CD4 counts >350, =350, =200, or very late in the infection when CD4 count is <100. Black dashed arrows indicate progression to AIDS while on treatment (treatment failure), meanwhile red dotted arrows indicate stopping treatment (drop out from ART).

HIV transmission occurs through sexual partnerships. Although these are instantaneous in the model, the influence of the variation in the relationship period is reflected by specifying the rate of infection through the partnership as a function of the number of sex acts in partnerships per year. That is, short-term partnerships effectively have a low rate of sex per year, whilst partnerships maintained over year would have a higher rate of sex per year. The probability of HIV transmission per sex act for those with chronic infection is based on the most recent meta-analysis , with the trend according to stage of infection based on the data from sero-discordant couples in Rakai Uganda . It is further assumed that the chance of male-to-female transmission is greater than the chance of male-to-female transmission by fixed multiplicative factor. Thus, infectiousness of an infected individual per sex act can depend whether he/she is a man or a woman, circumcised or not (if a man), on ART or not, and on the stage of his/her infection.

The impact of condom use is to reduce the chance of transmission in sex acts in which they are used. There are, thus, two parameters specifying the impact of condom use – (i) efficacy in preventing transmission in a sex act if they are used correctly; and,(ii) usage in sex acts (proportion of sex acts in a partnership, which can vary by partnership type). Usage changes over time to reflect the increase in condom use since 1998 indicated in national surveys and observational studies on sexual behaviour in South Africa .

The influence of circumcision is incorporated by dividing the male population into two parts - circumcised and uncircumcised men. Circumcised men are less likely to acquire HIV infection by a fixed multiplicative factor per sex act with infected women, and less likely to transmit infection by a fixed amount per sex act with uninfected women. The proportion of men in the model starting sex that enter the circumcised or uncircumcised groups corresponds to the proportion of men that are circumcised at birth or adolescence. The rate of movement from the uncircumcised to the circumcised group corresponds to the influence of an intervention providing adult male circumcision services.

The influence of testing is incorporated by introducing further stratifications that track individuals’ testing and behaviour change depending on whether the tested individual is infected or susceptible (Figure S2). For infected individuals four categories are tracked: never been tested and have baseline risk behavior, newly diagnosed with HIV and could change risk behavior, tested negative before then got infected and unaware of status, and finally diagnosed with HIV but has baseline risk behaviour. For susceptible individuals three categories are tracked and each is assumed to have baseline risk behavior: never been tested, tested once, and tested more than once. The “First test” rate in Figure S2 is indicative of “regular” testing efforts and moves baseline populations (highlighted by a blue gray oval) to “positive aware” and “negative aware” status according to HIV status whether it is positive or negative, respectively. With more intensive testing efforts (“Repeat test”) proportions of susceptible individuals who have been tested before will be tested again. As a consequence of awareness of status newly HIV diagnosed individuals could be set to reduce their risk behaviour by increasing condom use and/or decreasing rate of partnership formation for a certain duration after which they return back to their baseline risk behaviour before testing and these changes can be set marginal in those tested and find themselves not infected including if they got infected after testing (highlighted by a dark grey bubble in Figure S2).

Antiretroviral treatment (ART) can be initiated in multiple modes. Treatment can be initiated from any HIV stage or the moment individual’s CD4 count drops below 350, 200, or 100 cells/mm^3^. In Figure S1, “ART at CD4>350” refers to starting treatment from all HIV stages with CD4>350, while “ART at CD4=350” corresponds to starting treatment at CD4=350 and “Late ART” refers to starting treatment at CD4=200 and CD4=100 combined. ART initiation below CD4 count of 100 is used to represent the mode for actual ART initiation in recent years in South Africa . Thresholds of 350 and above for starting treatment apply only to infected individuals who are aware of their status due to testing. Meanwhile, individuals who are not tested are not put immediately on treatment the moment their CD4 count drops below 200 but after certain waiting time. No waiting time is assumed for individuals who are put on treatment when their CD4 drops below 100. The model incorporates dropping out treatment through drop out rates from all ART categories. Drop outs from ART categories “ART at CD4>350” and “Late ART” progress to ART=350 and AIDS after certain time intervals represented by the “Drop outs from Early ART” and “Drop Outs from Late ART” compartments in Figure S1. ART is assumed to extend the survivability of treated individuals while reducing onward transmission .


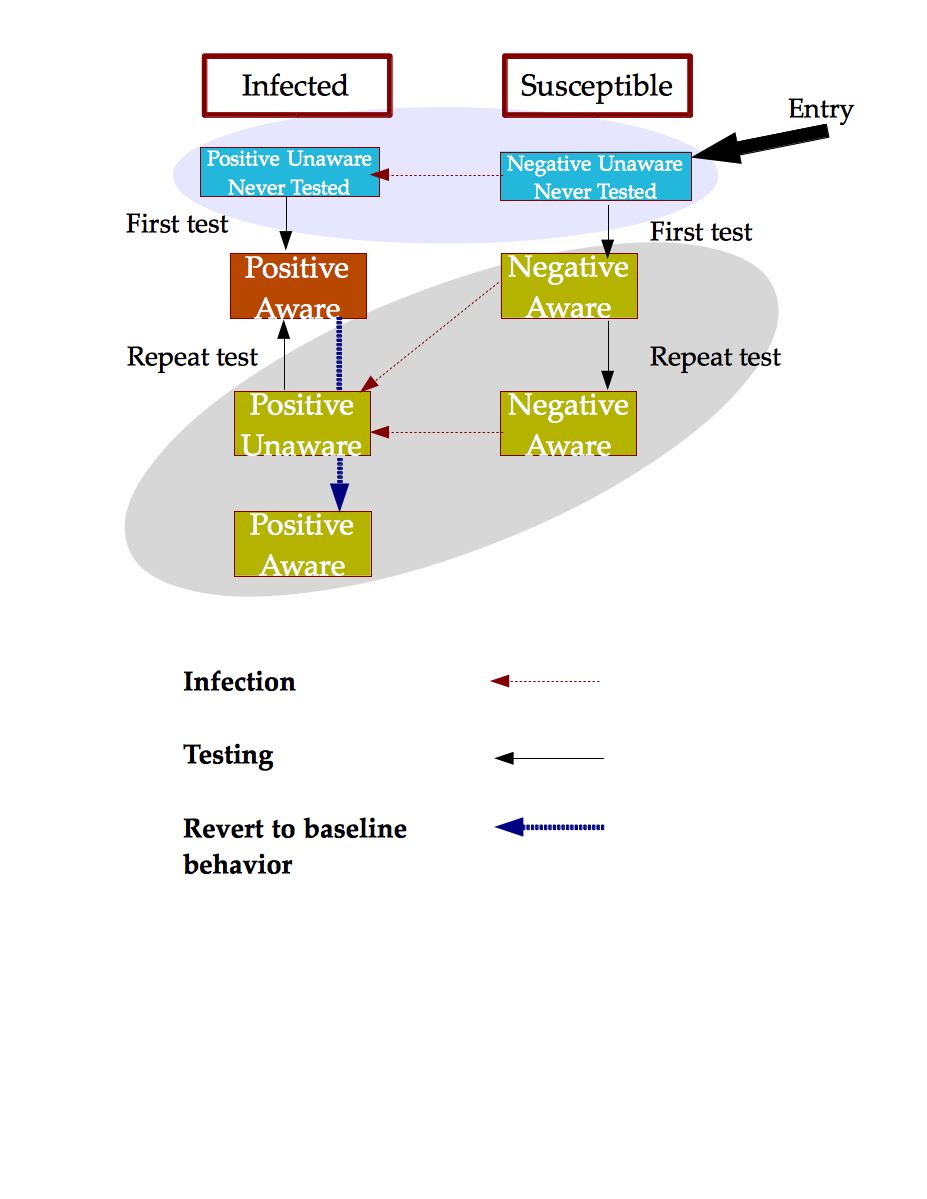


**Figure S2:** Flow diagram showing model representation of HIV testing categories. Infected and susceptible individuals have never been tested at baseline (light blue oval). Dark gray oval indicates all categories of individuals that have been tested before but have baseline sexual risk behavior either because they have no risk behavior change following testing or they have reverted back to baseline risk behavior after a mean duration of lower risk behavior.

**Model Fitting**

To calibrate model using data and epidemiological information we follow the Rejection Sampling approach to model fitting, which incorporates multiple sources of data in model parameter estimation and allows generating multiple model fits consistent with the available data and information. The data we used are: (1) KZN HIV prevalence data and its confidence intervals reported in population surveys 2002, 2004, 2005, and 2008 , (2) an estimated incidence and its confidence interval reported in 2004/2005 by the Africa Center for Health and Population Studies , (3) cumulative number of adults ever on ART reported by the Department of Health of Republic of South Africa , and (4) antenatal prevalence data reported by the same department . The multiple fits to data are obtained by allowing model parameters that are difficult to estimate reliably to be randomly selected from within some credible limits using assigned prior distributions (Table S1). For instance, because the HIV transmission probabilities and sexual behavior parameters are difficult to estimate reliably, prior distributions have been assigned to each of these parameters. The a priori selected model parameters forms a proposed combination set (or a proposed model fit) in which each parameter is represented by a single value from within its credible limits and used to generate model incidence and prevalence outputs. The set (or fit) is accepted (accepted model fit) or rejected depending whether or not it produces model estimates of prevalence and incidence within confidence intervals of data at the respective year. In each of the combination sets fixed values of parameters pertain to ART initiation for KZN are assumed because these values have been adjusted to generate the best fit to the number of people ever initiated ART in KZN. This procedure is repeated for a large number of proposed model fits until a substantial number of accepted model fits are obtained (Figure 1, main text). In the presence of an intervention a scenario is simulated in each of these accepted models and the estimated impact is then summarized over the produced impact values.

**Table S1: Prior distribution of model parameters**

| Parameter | Symbol | Prior distribution | Notes |
| --- | --- | --- | --- |
| Transmission probability from men to women in a single act of unprotected sex | $\beta_{0}$ | Triangular(0.0005,0.0015,0.0008) | Representative range captures impact of other risk factors such as other STIs not explicitly in the model |
| Degree of assortativeness in the mixing | $\varepsilon$ | Uniform(0.2,0.9) | Limited studies of sex partner network suggest a pattern of weak assortivity |
| Current degree of condom non-use during sex acts reached over a time span of 2-10 years ( a value of 1 corresponds to ceased condom use) | $\bar{q}$ | Uniform(0.28,1) | Based on reported levels of increase in condom use in the 2002-2008 South African National Surveys (HSRC) and that this increase has started around 1998 |
| Condom use in low risk activity groups | $\kappa_{L}$ | Uniform(0, 0.1) | Cannot be directly estimated from data |
| Condom use in medium risk activity groups | $\kappa_{M}$ | Uniform(0.05, 0.2) | Cannot be directly estimated from data |
| Condom use in high risk activity groups | $\kappa_{H}$ | Uniform(0.3, 0.6) | Cannot be directly estimated from data |
| Year condom use started to increase | $\xi$ | Uniform(1998, 2002) | Representative range based on what is reported by observational studies on sexual behavior and in the HSRC survey |
| Time span for increase in condom use | $\tau_{c}$ | Uniform(2,10) | Representative |
| New partner acquisition rate for low risk men | $c_{m}(1)$ | Uniform(0.01,1) | Representative |
| New partner acquisition rate for medium risk men | $c_{m}(2)$ | Uniform(1,10) | Representative |
| New partner acquisition rate for high risk men | $c_{m}(3)$ | Uniform(10,50) | Representative |
| New partner acquisition rate for low risk women | $c_{f}(1)$ | Uniform(0.01,1) | Representative |
| New partner acquisition rate for medium risk women | $c_{f}(2)$ | Uniform(1,5) | Representative |
| New partner acquisition rate for high risk women | $c_{f}(3)$ | Uniform(5,20) | Representative |
| Fraction of women in low risk group | $\psi_{f}(1)$ | Uniform(0.65,0.99) | Representative |
| Fraction of non-low risk women in the highest risk group | $\frac{\psi_{f}(3)}{1-\psi_{f}(1)}$ | Uniform(0.1,0.9) | Representative |
| Fraction of men not in low risk group | $\psi_{m}(1)$ | Uniform(0.6,0.99) | Representative |
| Fraction of non-low risk men in the highest risk group | $\frac{\psi_{m}(3)}{1-\psi_{m}(1)}$ | Uniform(0.2,0.9) | Representative |
| Year epidemic starts | $t_{0}$ | Uniform(1982,1987) | First AIDS cases are reported in early 1980s and data from US Census Bureau suggests that HIV started several years later in South Africa than it did in other Sub-Saharan African countries |
| Year ART starts | $t_{late}$ | Fixed(2004.5) | KZN Department of Health and Africa Center for Health and Population Studies |
| Scale up for access to ART at CD4<=200 | $a_{rate}$ | Fixed(0.50) | DOH data gives number of people ever started ART and the stated scale up rate is calibrated to fit model output for ever started ART to DOH data |
| Mean interval (years) before starting ART at CD4=200 for individuals unaware of their HIV-positive status | $\tau_{B}$ | Fixed(1) | To account for observed delay in starting ART in KZN . HIV testing is the root cause for this delay in Africa |

**Technical Specifications**

The model is a deterministic compartmental model defined by a set of ordinary differential equations that are solved numerically using custom-written software by the authors in Matlab 7.0. Model assumptions are listed in Table 2.

Untreated HIV pathogenesis

Susceptible individuals are at risk of acquiring HIV through heterosexual contacts with infected individuals and the hazards $\Lambda^{S}$ give the probability per unit time that a susceptible individual would get ever infected. Untreated HIV pathogenesis is represented by five compartments based on CD4 counts and development of symptoms: early acute stage ($Y_{A}$), when CD4 count is larger than 350 ($Y_{>350}$), when CD4 count is in the range 200-350 ($Y_{>200}$), interval when CD4 count is below 200 and the infected individual remains asymptomatic ($Y_{L}$), and interval when the infection has worsen and symptoms start to develop with high viral load ($Y_{H}$), and full blown AIDS ($A$). Untreated HIV infection is specified by the following ordinary differential equations:

$$\begin{matrix} \frac{dS(l,k,v)}{dt}=\left(\mu+\alpha\right)N\Psi-\mu S-\Lambda^{S}S \\ \frac{dY_{A}(l,k,v)}{dt}=\Lambda^{S}S-\mu Y_{A}-\sigma_{1}Y_{A} \\ \frac{dY_{>350}(l,k,v)}{dt}=\sigma_{1}Y_{A}-\mu Y_{>350}-\sigma_{2}Y_{>350} \\ \frac{dY_{>200}(l,k,v)}{dt}=\sigma_{2}Y_{>350}-\mu Y_{>200}-\sigma_{3}Y_{>200} \\ \frac{dY_{L}(l,k,v)}{dt}=\sigma_{3}Y_{>200}-\mu Y_{L}-\sigma_{4}Y_{L} \\ \frac{dY_{H}(l,k,v)}{dt}=\sigma_{4}Y_{L}-\mu Y_{H}-\sigma_{5}Y_{H} \\ \frac{dA(l,k,v)}{dt}=\sigma_{5}Y_{H}-\mu A-\gamma A\text{ } \end{matrix}$$

Where each of the above compartments is stratified further by the indices which are suppressed for conciseness in the right hand sides of equations . Stratification given by index $k$is to females ($k$=1), uncircumcised males ($k$=2), and circumcised males ($k$=3). To capture the heterogeneity in the number of sexual partners, men and women in the model were stratified into risk groups that form different numbers of sexual partnerships. Those in the higher risk groups tend to form more partnerships, but each of these partnerships comprises fewer sex acts and higher condom use. Stratification given by index $l$ is based on “risk activity” defined by the average number of sexual partners per year and it is to low ($l$=1), intermediate ($l$=2), and high ($l$=3) risk, respectively. Stratification according to $v$ tracks individuals’ testing and behaviour change categories according to whether the individual is infected or susceptible. For infected individuals index values$v$=1,2,3, and 4 categorizes individuals that have never been tested and have baseline risk behaviour (baseline category highlighted by light blue oval, Figure S2), that are aware of status with the possibility of conforming to a change in risk behaviour (red box, Figure S2), that think uninfected and have baseline risk behaviours (if infected after being tested negative), and individuals that are aware of status and have baseline risk behaviours. For susceptible individuals $v$=1 indicate individuals that have never been tested while the categories ($v$=2 and 3) indicate individuals that have baseline risk behaviour and have been tested once and more than once, respectively.

Intervals for a given infection stage are given by the $\sigma_{s}\text{ (}s=1\text{, }2,\text{ ..., }5)$ rates (1/interval spent in that stage). Infectiousness of an infected individual can depend whether he/she is a man or a woman, circumcised or not (if a man), on ART or not, and on the stage of his/her infection. AIDS mortality is captured by the parameter $\gamma$ while the parameter $\mu$ is a fixed demographic birth and death rate assumed by the model.

Demography

At each instant of time the entry to the sexually active population is specified by the term $\left(\mu+\alpha\right)N\Psi$ in equation . The $\Psi(l,k,v)$ is the matrix of population distribution over each $l,k,v$ stratum and it is given in terms of $\psi_{f}\text{ and }\psi_{m}$ which are the fraction of females and males in each risk activity group. The matrix$\Psi(l,k,v)$ is given by:

The parameter $f_{cm}$ gives the fraction of males circumcised at adolescence. The model does not stratify the population explicitly according to age. A fixed intrinsic growth rate of $\alpha$ is assumed for the sexually active population. The parameter $N$ in equation gives the total number of the population.

Interventions

*Testing and behavior change:* Testing and retesting is implemented as in Figure S2 by movements between categories specified by index $v$ as in the following equations (other indices are suppressed but are implicitly assumed):

$$\begin{matrix} \frac{dS(v=1)}{dt}=\frac{dS(v=1)}{dt}-\eta_{T}S(v=1) \\ \frac{dS(v=2)}{dt}=\frac{dS(v=2)}{dt}+\eta_{T}S(v=1)-\eta_{T}S(v=2) \\ \frac{dS(v=3)}{dt}=\frac{dS(v=3)}{dt}+\eta_{T}S(v=2) \\ \frac{dI(v=1)}{dt}=\frac{dI(v=1)}{dt}-\eta_{T}I(v=1) \\ \frac{dI(v=3)}{dt}=\frac{dI(v=3)}{dt}-\eta_{T}I(v=3) \\ \frac{dI(v=2)}{dt}=\frac{dI(v=2)}{dt}+\eta_{T}I(v=1)+\eta_{T}I(v=3)-\frac{1}{\tau_{T}}I(v=2) \\ \frac{dI(v=4)}{dt}=\frac{dI(v=4)}{dt}+\frac{1}{\tau_{T}}I(v=2) \end{matrix}$$

where the existence of derivatives on both sides of the equation indicates updating previous derivatives in Eq. for testing, $I$ represents any of the five infection stages before AIDS in Eq. , $\eta_{T}$ is the testing and retesting rate, and $\tau_{T}$ is the average time during which a tested individual would have a change in risk behaviour (risk reduction following first HIV diagnosis) and after which reverts back to baseline risk behaviour. It is worth mentioning that in our model’s calculations presented in main text we have not assumed any behaviour change undertaken by HIV-negative individuals upon knowing status. Individuals who initiate ART upon knowledge of status are assumed to flow from category $v=2$ to category $v=4$ with a rate equal to $\frac{1}{\tau_{T}}$to account for the waning of their behaviour change after knowledge of status.

*Antiretroviral treatment (ART):* ART can be initiated either immediately upon knowing infection status (categories $v=2,4$) from any stage of infection including when CD4<=200 or without knowing status and only from stages of infection with CD4≤200. Individuals on treatment could stop treatment altogether (drop out) or the treatment that they adhere to could fail. The following equations represent model implantation of initiation of late ART at CD4≤200 for all testing categories ($v=1,2,3,4$):

where the existence of the derivative on both sides indicates updating previous HIV equations (c.f. Eq. and Eq. ) by incorporating ART. Here $a_{200}$ is the parameter that gives scale up for access to ART at CD4=200 according to testing category, $a_{50}$ is the ART scale up parameter at CD4<100 according to testing category,$B_{200}$ is a compartment representing the delay time ($\tau_{B}$) spent before starting ART at CD4=200 which depends on testing category, $T_{\leq200}$ represents individuals on ART that have initiated ART at CD4≤200, $D_{\leq200}$ represents individuals who stopped treatment from $T_{\leq200}$ (at a rate given by$\delta_{200}$), $\varpi_{\leq200}$ represents rate of progression from $D_{\leq200}$to AIDS, and $\omega_{\leq200}$ represents the rate of treatment failure and progression to AIDS from $T_{\leq200}$. The parameters $a_{200}$, $a_{50}$, and $\tau_{B}$ can be set for individuals who know their status ($v=2,4$) differently. For example a much smaller delay time ($\tau_{B}$) spent in the compartment $B_{200}$ is assumed when treatment at CD4=200 is initiated with knowledge of status. Initiating treatment at CD4≤200 based on knowledge of status (categories $v=2,4$) is represented by updating parameters in Eq. and initiating treatment at CD4>200 is assumed only based on knowledge of status and is implemented by the following equations for categories $v=2,4$:

$$\begin{matrix} \frac{dY_{A}}{dt}=\frac{dY_{A}}{dt}-a_{>350}Y_{A} \\ \frac{dY_{>350}}{dt}=\frac{dY_{>350}}{dt}-a_{>350}Y_{>350} \\ \frac{dT_{>350}}{dt}=a_{>350}Y_{A}+a_{>350}Y_{>350}-\mu T_{>350}-\omega_{>200}T_{>350}-\delta_{>200}T_{>350}\text{ } \\ \frac{dD_{>350}}{dt}=\delta_{>350}T_{>350}-\mu D_{>350}-\varpi_{>350}D_{>350}\text{ } \\ \frac{dY_{>200}}{dt}=\frac{dY_{>200}}{dt}-a_{350}\sigma_{2}Y_{>350}+(1-a_{350})\varpi_{>350}D_{>350}\text{+ }\delta_{>200}T_{350} \\ \frac{dT_{350}}{dt}=a_{350}\sigma_{2}Y_{>350}+a_{350}\varpi_{>350}D_{>350}-\mu T_{350}-\omega_{>200}T_{350}-\delta_{>200}T_{350} \\ \frac{dA}{dt}=\frac{dA}{dt}+\omega_{>200}T_{>350}+\omega_{>200}T_{350} \end{matrix}$$

here the compartments$T_{>350}$, $D_{>350}$, and $T_{350}$ represents individuals who have initiated treatment at CD4>350, who have left $T_{>350}$ compartment by stopping treatment, and who have initiated treatment at CD4=350, respectively. The parameters $a_{>350}$, $\delta_{>200}$, $\varpi_{>350}$, $a_{350}$, and $\omega_{>200}$ represent rate of scale up for initiating ART at CD4>350 (including from acute infection), rate of leaving early ART (initiated at CD4>200) by stopping treatment, rate of disease progression to CD4=350 after stopping treatment that was initiated at CD4>350, and rate of treatment failure and disease progression to AIDS from ART starting at CD4>200. The existence of derivatives in both sides of some equations indicates updating previous equations.

*Circumcision:* is implemented in the model as an intervention that could be provided for both susceptible and infected men by moving men at certain time from category ($k=2$) to category ($k=3$) using scale up parameter $\eta_{C}$ as in the following equations (all other indices are suppressed but implicitly assumed):

$$\begin{matrix} \frac{dS(k=2)}{dt}=\frac{dS(k=2)}{dt}-\eta_{C}S(k=2) \\ \frac{dS(k=3)}{dt}=\frac{dS(k=3)}{dt}+\eta_{C}S(k=2) \\ \frac{dϒ(k=2)}{dt}=\frac{dϒ(k=2)}{dt}-\eta_{C}ϒ(k=2) \\ \frac{dϒ(k=3)}{dt}=\frac{dϒ(k=3)}{dt}+\eta_{C}ϒ(k=2) \end{matrix}$$

here $ϒ$ is a generic symbol representing any infection stage including ART categories and drop outs from ART. Although the model incorporates circumcision effect on acquisition and transmission, only acquisition effect has been used in the calculations of the main text.

Infection and sexual network

As mentioned before the $\Lambda^{S(l,k,v)}$are the average HIV forces of infection (hazard rates of infection) at a given time experienced by each individual in the heterosexual susceptible population $S(l,k,v)$ from the pool of infected population of the opposite sex mixing with that individual. These hazard rates generally depend on the rates of change of sexual partners, proportion of the infected population from the opposite sex in each risk category, on the mixing pattern between different risk groups, and on the likelihood of transmission per partnership ($z$). The likelihood of transmission per partnership depends on the frequency of sex acts, on the probability of transmission per coital act (generally denoted by$\beta$), on the degree of condom use in the partnership, and whether any prevention intervention such as circumcision or ART is being consumed by the partners. We assume a baseline transmission probability $\beta_{0}$ in the lack of ART from uncircumcised males with chronic infection to females. Therefore, the baseline transmission probability per coital act from females with no ART to uncircumcised males would be $\beta_{females}\times\beta_{0}$ where $\beta_{females}$is the factor change in transmission probability per sex act from females to males. Similarly we incorporate the impact on transmission or acquisition per sexual act of each stratum with respect to baseline using a multiplicative factor. For example $\beta_{Circ}^{Acq}$ denotes the reduction ratio with respect to baseline in transmission probability per coital act from females to circumcised males; thus $\beta_{Circ}^{Acq}\times\beta_{females}\times\beta_{0}$ gives the probability of transmission from females with chronic infection to circumcised males. Let $ϒ(l,k,v)$ stands for any of the HIV infected compartments irrespective to infection stage or treatment status (e.g. $ϒ(l,k,v)$ could be =$Y_{>350}$, or $ϒ(l,k,v)$ could be =$T_{350}$) , then for illustration the likelihoods of transmission per partnership between females and circumcised males and vice versa are given by the binomial probabilities:

$$\begin{matrix} z_{ϒ\left(l',1,v'\right)\to S(l,3,v)}=1-(1-\beta_{Circ}^{Acq}\beta_{females}\beta_{ϒ\left(l',1,v'\right)}\beta_{0})^{\left(1-R_{v'}^{c}R_{v}^{c}(1-\bar{Q}_{t}NU_{l,l'})\right)n_{sex}(l,l')}\times\\ \text{ }(1-\beta_{Circ}^{Acq}\beta_{females}\beta_{ϒ\left(l',1,v'\right)}\beta_{condom}\beta_{0})^{R_{v'}^{c}R_{v}^{c}(1-\bar{Q}_{t}NU_{l,l'})n_{sex}(l,l')}, \\ z_{ϒ(l,3,v)\to S\left(l',1,v'\right)}=1-(1-\beta_{Circ}^{Tra}\beta_{ϒ(l,3,v)}\beta_{0})^{\left(1-R_{v'}^{c}R_{v}^{c}(1-\bar{Q}_{t}NU_{l,l'})\right)n_{sex}(l,l')}\times\\ \text{ }(1-\beta_{Circ}^{Tra}\beta_{ϒ(l,3,v)}\beta_{condom}\beta_{0})^{R_{v'}^{c}R_{v}^{c}(1-\bar{Q}_{t}NU_{l,l'})n_{sex}(l,l')} \end{matrix}$$

the parameters $\beta_{ϒ(l,k,v)}$ is introduced to account for variation in transmission probability by stage of infection and/or treatment status and gives the factor change in transmission probability per coital act from the infected compartment $ϒ$. The quantities denote probability to avoid condom use in a sex act between two individuals unaware of their infection status and of risk categories $l$and $l'$before some calendar time $\xi$ after which reported condom usage started to increase. The quantity scales ( such that $\bar{Q}_{t}NU_{l,l'}$<=1) to reflect increase in condom use in the population at calendar time $t$> $\xi$:

where $\bar{q}$is the current degree of avoiding condom use reached over a duration $\tau_{c}$ (see Table S1). The number of unprotected sex acts in a partnership between individuals of risk categories $l$and $l'$ is $n_{next}(l,l')$.The knowledge of HIV status affects condom use in partnerships between individuals via the relative ratios $R_{v}^{c}$ and $R_{v'}^{c}$ which give the relative increase in condom use for each partner according to whether they have been tested HIV-positive or otherwise no effect ($R_{1}^{c}=1$ by definition).

After introducing the likelihoods of transmission per partnership between males and females we introduce the number of partnerships $W_{m}^{ϒ}(l)$that a susceptible male of risk $l$ will form with infected females in the generic compartment $ϒ$and risk $l'$:

$$\begin{matrix} W_{m}^{ϒ}(l)=c_{m}(l)G_{f\to m}(l,l')\times\frac{ϒ\left(l',1,v'\right)}{N_{f}(l')}, \\ G_{f\to m}(l,l')=\varepsilon\delta_{ll'}+(1-\varepsilon)\frac{c_{f}(l')N_{f}(l')}{\sum_{l''} c_{f}(l'')N_{f}(l'')} \end{matrix}$$

where $N_{f}(l')$ is the total number of females with risk category$l'$. The parameters $c_{f}(l')$ and $c_{m}(l)$ respectively give the mean number of partners in a year per female and male in each activity group (rates of change of sexual partner), while $G_{f\to m}(l,l')$ gives the mixing pattern between the two risk activity groups. Meanwhile the degree of assortativeness in mixing is given by $\varepsilon$. This makes us ready to introduce the hazard rates of infection, by multiplying the number of partnerships per male $W_{m}^{ϒ}(l)$ and the likelihood of transmission per partnership and summing over all compartments of infected females and strata and by doing the same corresponding steps for females we get for males and females, respectively:

$$\begin{matrix} \Lambda^{S(l,k,v)}=\sum_{l';v';ϒ} R_{v}^{p}R_{v'}^{p}\times W_{_{m}}^{ϒ\left(l',1,v'\right)}(l)\times z_{ϒ\left(l',1,v'\right)\to S(l,k,v)}\text{; }k=2,3 \\ \Lambda^{S(l',1,v')}=\sum_{l;k=2,3;v;ϒ} R_{v'}^{p}R_{v}^{p}\times W_{_{f}}^{ϒ(l,k,v)}(l)\times z_{ϒ(l,k,v)\to S(l',1,v')} \end{matrix}$$

Where the quantities $R_{v}^{p}$and $R_{v'}^{p}$ give the relative decrease in partner acquisition rates for the individuals brought in by whether they become aware of their HIV-positive status or otherwise no effect ($R_{1}^{p}=1$ by definition). We impose a male-driven balance in the number of partnerships such that if males increase their risk behaviour by increasing their new partnership acquisition rate; this translates into an increase in the level of risk behaviour experienced by females. This balance is represented in the equation:

$$G_{m\to f}(l',l)=G_{f\to m}(l,l')\times\frac{c_{m}(l)N_{m}(l)}{c_{f}(l')N_{f}(l')}$$

**Table S2: Model assumptions**

| Assumption | Parameter value | Sources |
| --- | --- | --- |
| Demography and sexual behaviour | | |
| Average sexual life span | 35 years | Representative of adult population of age (15-49) |
| Initial population size ($N_{0}$) | $7.6\times10^{6}$ | With the assumed population growth the population size in 2010 is about $10.6\times10^{6}$ |
| The intrinsic population growth rate ($\alpha$) | 0.02 | Projected population growth rate for SA estimated between October 2001 and 2006 is 0.7% based on the Stats SA med-year estimates over 4.75 years . The parameter is set to give this growth rate and KZN population in 2010 reported in census |
| Number of unprotected sexual acts per partnership between individuals in different risk activity groups: |  |  |
| In a partnership with low sexual activity individual | 100 | Estimated based on estimated frequency of sex in marital relationships in South Africa |
| Otherwise | 2 |  |
| Untreated HIV pathogenesis | | |
| Duration of acute infection ($1/\sigma_{1}$) | 0.25 years |  |
| Mean duration from CD4 >350 to CD4=350 ($1/\sigma_{2}$) | 4.6 years | Representative |
| Mean duration from CD4=350 to CD4=200 ($1/\sigma_{3}$) | 3.5 years | Representative |
| Mean duration from CD4 =200 to CD4=100 ($1/\sigma_{4}$) | 1.1 years | Representative |
| Mean duration of heightened infectiousness due to sudden increase in viral load before AIDS ($1/\sigma_{5}$) | 10 months |  |
| Survival time with AIDS ($1/\gamma$) | 0.8 years |  |
| Multiplicative factor change in baseline transmission probability ($\beta_{0}$) | | |
| From population with acute infection ($\beta_{Y_{A}}$) | 27 |  |
| From females relative to from males ($\beta_{females}$) | 0.5 |  |
| From HIV infected population at risk of opportunistic infections and heightened viremia in late symptomatic infection stages ($Y_{H}$ population)($\beta_{Y_{H}}$) | 7 |  |
| From individuals with AIDS ($A$ population) ($\beta_{A}$) | 0 | Estimated |
| From circumcised men ($\beta_{\text{Circ}}^{\text{Tra}}$) | 1 | No effect |
| From individuals on ART ($\beta_{\text{ART}}^{\text{Tra}}$) | 0.08 (Corresponds to efficacy in reducing infectivity by 92%) |  |
| To circumcised men ($\beta_{\text{Circ}}^{\text{Acq}}$) | 0.4 (Corresponds to efficacy in reducing susceptibility by 60%) |  |
| Condoms both ways | 0.1 |  |
| Existing levels of interventions | | |
| Proportion of eligible adults that are tested per year | 20% |  |
| Change in risk behavior following HIV-positive test | | |
| Increase in condom use (men/women) | 12.5%/6.25% | Representative values |
| Decrease in partner acquisition rate (men/women) | 12.5%/12.5% | Representative values |
| Mean duration of keeping the above behaviour changes ($\tau_{T}$) | 1 year | Representative value |
| Fraction of males circumcised at adolescence ($f_{cm}$) | 0.27 |  |
| Proportion initiating treatment at CD4≤200 | 52% |  |
| Average delay time before initiating treatment at CD4>100 (unaware of status) ($\tau_{B}(v),\text{ }v=1,3$) | 1 year |  |
| Average delay time before initiating treatment at CD4>100 (aware of status) ($\tau_{B}(v),\text{ }v=2,4$) | No delay (immediate treatment) |  |
| Rate of drop out from ART initiated at any CD4 count ($\delta$) | 14.5% per year |  |

Table S3: Assumed percentage of sex acts in heterosexual partnerships in which condoms were used (baseline values before the start of the modeled intervention). The credible limits between the parentheses reflect the uncertainty in model fitting.

|  | Males | | | |
| --- | --- | --- | --- | --- |
| Females | Sexual Activity Group | Low | Medium | High |
|  | Low | 29% (19%-41%) | 29% (19%-41%) | 32% (23%-44%) |
|  | Medium | 29% (19%-41%) | 29% (19%-41%) | 32% (23%-44%) |
|  | High | 32% (23%-44%) | 32% (23%-44%) | 64% (59%-70%) |

**
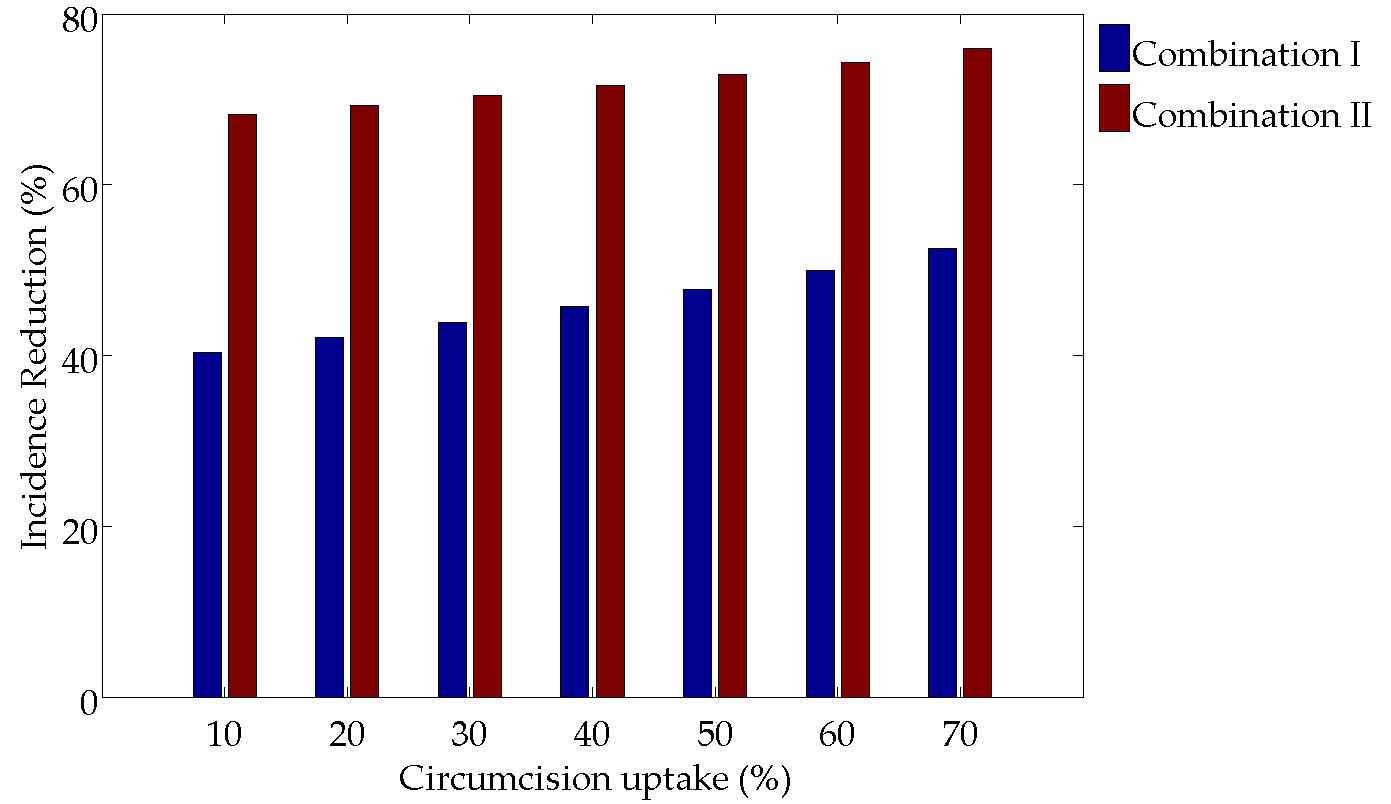
**

**Figure S3:** The sensitivity of the impact of Combination I and Combination II interventions at year 15 to the amount of circumcision uptake. In the main text, circumcision uptake in these two interventions is assumed 70%.

**References**
